# Magnetic Resonance Comparison of Left-Right Heart Volumetric and Functional Parameters in Thalassemia Major and Thalassemia Intermedia Patients

**DOI:** 10.1155/2015/857642

**Published:** 2015-04-02

**Authors:** Carlo Liguori, Francesca Pitocco, Ilenia Di Giampietro, Aldo Eros De Vivo, Emiliano Schena, Francesco Giurazza, Francesco Sorrentino, Bruno Beomonte Zobel

**Affiliations:** ^1^Department of Radiology, A.O.R.N. Cardarelli, Via Antonio Cardarelli 9, 80100 Naples, Italy; ^2^Department of Diagnostic Imaging, Campus Bio-Medico University, Via Alvaro del Portillo 200, 00128 Rome, Italy; ^3^Unit of Measurements and Biomedical Instrumentation, Campus Bio-Medico University, Via Alvaro del Portillo 200, 00128 Rome, Italy; ^4^Department of Hematology, Sant'Eugenio Hospital, Piazzale dell'Umanesimo 10, 00143 Rome, Italy

## Abstract

*Objectives*. To evaluate a population of asymptomatic thalassemia major (TM) and thalassemia intermedia (TI) patients using cardiovascular magnetic resonance (CMR). We supposed that TI group could be differentiated from the TM group based on *T*2^**∗**^ and that the TI group could demonstrate higher cardiac output. *Methods*. A retrospective analysis of 242 patients with TM and TI was performed (132 males, 110 females; mean age 39.6 ± 8 years; 186 TM, 56 TI). Iron load was assessed by *T*2^**∗**^ measurements; volumetric functions were analyzed using steady-state-free precession sequences. *Results*. Significant difference in left-right heart performance was observed between TM with iron overload and TI patients and between TM with iron overload and TM without iron overload (*P* < 0.05); no significant differences were observed between TM without iron overload and TI patients. A significant correlation was observed between *T*2^**∗**^ and ejection fraction of right ventricle- (RV-) ejection fraction of left ventricle (LV); an inverse correlation was present among *T*2^**∗**^ values and end-diastolic volume of LV, end-systolic volume of LV, stroke volume of LV, end-diastolic volume of RV, end-systolic volume of RV, and stroke volume of RV. *Conclusions*. CMR is a leading approach for cardiac risk evaluation of TM and TI patients.

## 1. Introduction

### 1.1. Physiopathological Background

*β*-Thalassemia is an inherited single gene disorder related to impaired synthesis of the beta globin chain of haemoglobin leading to defective *β*-chain production, an imbalance in *α*/*β* globin chain synthesis, ineffective erythropoiesis, and anaemia. Chronic haemolytic anaemia, resulting from ineffective erythropoiesis, is the hallmark of all thalassemia syndromes. The two ends of the broad spectrum of *β*-thalassemia range from asymptomatic carriers to patients who require lifelong transfusion related to a severe chronic anaemia in order to prolong survival and allow normal development [1, 2]. Depending on disease severity, two clinical forms of *β*-thalassemia are distinguished: thalassemia major (TM) and thalassemia intermedia (TI). TM is fatal unless adequate transfusions are started early, in conjunction with an intensive iron chelation therapy. TI is a clinical condition of intermediate severity between TM, thalassemia minor, and asymptomatic carrier [3]. TM and TI patients are exposed to prolonged tissue hypoxia that can lead to the development of bone marrow expansion, increasingly ineffective erythropoiesis, and increased intestinal iron absorption. Chronic anaemia contributes also to increase patients' susceptibility to infections and to a condition of hypercoagulability [4, 5].

Chronic haemolysis and iron overload, both present in case of haemoglobinopathies, are currently considered sources of strong oxidative stress. Reports [6, 7] have shown that the free heme and the red cell membrane elements that are produced during haemolysis have a negative effect on nitric oxide and arginine availability, which in turn promotes vasoconstriction. They can also lead to further endothelial dysfunction, resulting in a more pronounced nitric oxide reduction, as well as to diffuse elastic tissue injury. The presence of elastic tissue defect has been recently described with a high prevalence in patients with haemoglobinopathies, especially in those with either of the two forms of *β*-thalassemia. Lifelong blood transfusions lead to iron overload and toxicity, resulting in severe endocrine, liver, and cardiac dysfunctions [6–9]. Although TM and TI present some common basic pathophysiologic mechanisms, the cardiac involvement is different [5]. A high cardiac output following chronic tissue hypoxia and increased pulmonary and systemic vascular resistance represent the main causes of heart disease in TI. Pulmonary hypertension is a primary manifestation of heart involvement especially in right-sided failure in patients with TI [10]. Despite recent improvements in patient care, iron overload cardiomyopathy is still a leading cause of death in TM patients [11]. Although iron overload is mainly a problem for transfusion-dependent TM patients, it can also involve TI patients [12]. Thus, the early detection of myocardial iron-related cardiac toxicity is mandatory for the correct clinical management of TM and TI patients.

### 1.2. Role of Imaging

Cardiac magnetic resonance (CMR) has recently emerged as an important tool to noninvasively quantify cardiac iron load. When overloaded tissues are exposed to a magnetic field, the presence of iron causes concentration-related signal loss. While *T*1 relaxation time decreases moderately, *T*2 and *T*2^∗^ relaxation times have a significant decrease. *T*2^∗^ varies inversely with iron concentration because iron interferes with local magnetic field homogeneity and accelerates transverse signal decay.

*T*2^∗^ cardiovascular MR imaging with a single slice approach has been validated as a quantitative method to evaluate myocardial iron overload.

An Italian cooperative study showed that multislice multiecho *T*2^∗^ MRI provides a noninvasive, fast, reproducible means of assessing the heterogeneous distribution of myocardial iron overload, presenting a good correlation with the single septal *T*2^∗^ measurement technique [13–15]. Although cardiac pathophysiology has been deeply studied in TM patients, only few data are available about cardiac function in TI. We supposed that it is possible to differentiate the TI group from the TM group on *T*2^∗^ values.

Thus, the aims of this study were to evaluate the myocardial iron load and the left-right heart performance in an asymptomatic population of TM and TI patients using CMR. Our hypothesis was that the TI group could be differentiated from the TM group based on *T*2^∗^ values and that the TI patients would demonstrate significantly higher cardiac output compared to the TM population.

## 2. Materials and Methods

### 2.1. Patients Population

A total of 242 patients (132 males and 110 females; mean age 39.6+/+ 8 years; 186 TM, 56 TI; body surface area (kg/m^2^) 1.6 +/− 0.2) underwent CMR examinations. No patients presented clinical signs of cardiac failure according to Framingham's study criteria and NYHA criteria [16] and none had a history of pulmonary hypertension. No patients presented clinical and laboratory criteria for diabetes according to American Diabetes Association criteria (fasting blood sugar test: plasma glucose < 100 mg/dL; oral glucose tolerance test at 120 minutes: plasma glucose < 140 mg/dL) [17]. No nutritional deficiencies (including selenium, thiamine, vitamin D, and carnitine) and thyroid disease were diagnosed in our thalassemia population. Exclusion criteria were the coexistence of other cardiopulmonary or systemic diseases. TM patients were regularly transfused (12–20 blood transfusions/year) to maintain haemoglobin levels at 10 mg/dL. All TM patients were under iron chelation therapy with a desferrioxamine infusion, an oral chelator (deferiprone or deferasirox), or combination therapy of desferrioxamine and deferiprone. TI patients were occasionally transfused (0–6 blood transfusions/year and 93% of patients had at least 1 blood transfusion/year) and presented mean haemoglobin levels at 7–11 mg/dL. TI patients occasionally received chelation therapy in the form of desferrioxamine infusion. MRI evaluation was performed 48 hours after the last blood transfusion in TM patients. The local ethics committee approved the study and the patients gave their informed consent.

### 2.2. Imaging Techniques

All MR examinations were performed using a 1.5-Tesla scanner (Avanto, Siemens, Erlangen, Germany). To assess heart *T*2^∗^, a short-axis midventricular cardiac gated gradient multiecho dark blood single breath-hold sequence was acquired at eight echo times (TE = 1,56–17 ms) and a slice thickness of 10 mm. A delay time of 0 msec after the R-wave trigger was chosen to obtain high quality image reducing blood flow and myocardial wall motion artefacts. For analysis, a homogeneous full-thickness region of interest (ROI) was chosen in the interventricular septum that surrounded both epicardial and endocardial borders. The signal intensity of this region was assessed for each image and plotted against the TE to form an exponential decay curve using dedicated software (CMRtools; Cardiovascular Imaging Solutions, London, UK). To obtain *T*2^∗^, an exponential trend line was fitted with an equation in the form *y* = *Ke* ^−TE/*T*2^∗^^, where *K* is a constant and *y* is the image signal intensity. The lower limit of normality for *T*2^∗^ in the assessment of myocardial iron load has been considered 20 msec [12]. Patients with *T*2^∗^ > 20 msec were considered to be free of cardiac iron overload, while patients with *T*2^∗^ < 20 msec were considered to have cardiac overload. To assess left and right ventricles volumes and functions, breath-hold short-axis slices from the base to apex were acquired with a 10 mm slice thickness and no gap, using steady-state-free precession (SSFP) sequence. Semiautomated software (CMRtools; Cardiovascular Imaging Solutions, London, UK) was used to assess the left ventricle end-diastolic volumes (EDVLV) and left ventricle end-systolic volumes (ESVLV), left ventricle stroke volume (SVLV), left ventricle ejection fraction (EFLV), right ventricle end-diastolic volumes (EDVRV) and right ventricle end-systolic volumes (ESVRV), right ventricle stroke volume (SVRV), right ventricle ejection fraction (EFRV), and left-right ventricular masses. The epicardial and endocardial borders were outlined during the cardiac cycle in the short-axis slices; then the mitral, tricuspid, aortic, and pulmonary valves planes were tracked, to correct for alteration in volume due to the descent of the atrioventricular ring toward the apex during systole. Since the body habitus may be below average in TM patients, all parameters were indexed to the body surface area, calculated using the Mosteller formula (*m* ^2^ = [(height (cm) × weight (kg)/3600)]^1/2^) from the patients' height and weight at the time of the MR examination [18].

### 2.3. Statistical Analysis

Simple regression analysis was performed to evaluate the correlation between *T*2^∗^ and the following parameters: EDVLVi, EDVRVi, EFLV, EFRV, ESVLVi, ESVRVi, SVLVi, SVRVi, and left-right ventricular masses. Correlation analysis was performed by Spearman rank correlation because the values of the mentioned parameters are not normally distributed (as confirmed by performing the one-sample Kolmogorov-Smirnov test). The Spearman rank correlation coefficient (*r*) and the significance of the simple regressions (*ρ*) were calculated. Furthermore, we evaluated the differences between TM and TI patients in terms of EDVLVi, EDVRVi, EFLV, EFRV, SVLVi, SVRVi, ESVLVi, and ESVRVi. The differences were analyzed using the Wilcoxon signed-rank test (*P* < 0.05). All the statistics were developed in the MATLAB (MathWorks, Inc.) environment. Statistical significance was considered for *P* < 0.05.

## 3. Results

**Figure 1:**
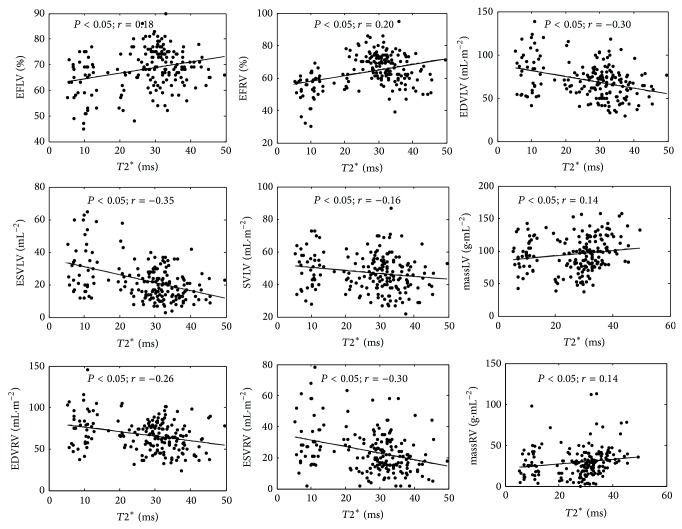
Simple regression analysis with *T*2^∗^ of the parameters of interest (i.e., EFLV, EFRV, MassLVi, MassRVi, EDVLVi, ESVLVi, SVLV, EDVRVi, and ESVRVi) in the cohort of the TM patients.

41 TM patients presented myocardial iron overload with *T*2^∗^ values < 20 ms. All TI patients (*n* = 56) presented no myocardial overload having *T*2^∗^ values > 20 ms. In TM patients (*n* = 186), a significant direct correlation was observed between *T*2^∗^ values and EFLV, EFRV, and masses, while a significant inverse correlation was observed among *T*2^∗^ values and EDVLVi, ESVLVi, SVLVi, EDVRVi, ESVRVi, and SVRVi (Figure 1). According to Marsella et al. [19], cardiac *T*2^∗^ was significantly lower in patients with heart dysfunction without differences according to sex.

**Figure 2:**
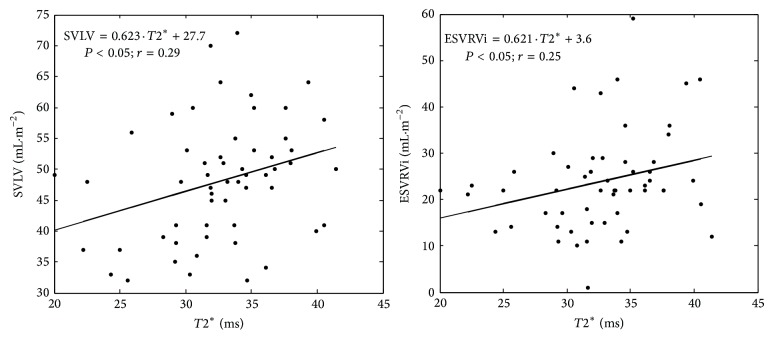
Simple regression analysis with *T*2^∗^ of SVLVi and ESVRVi in the cohort of the intermedia patients; the linear relationship is also shown.

TI patients presented significant direct correlations between *T*2^∗^ and SVLVi (0.623 × *T*2^∗^ + 27.7) and *T*2^∗^ and ESVRVi (0.621 × *T*2^∗^ + 3.6) (Figure 2). The further analysis was performed considering three groups: (*i*) TM patients with iron overload (*T*2^∗^ < 20 ms; *n* = 41); (*ii*) TM patients without iron overload (*T*2^∗^ > 20 ms; *n* = 145); (*iii*) TI patients (*n* = 56).

**Figure 3:**
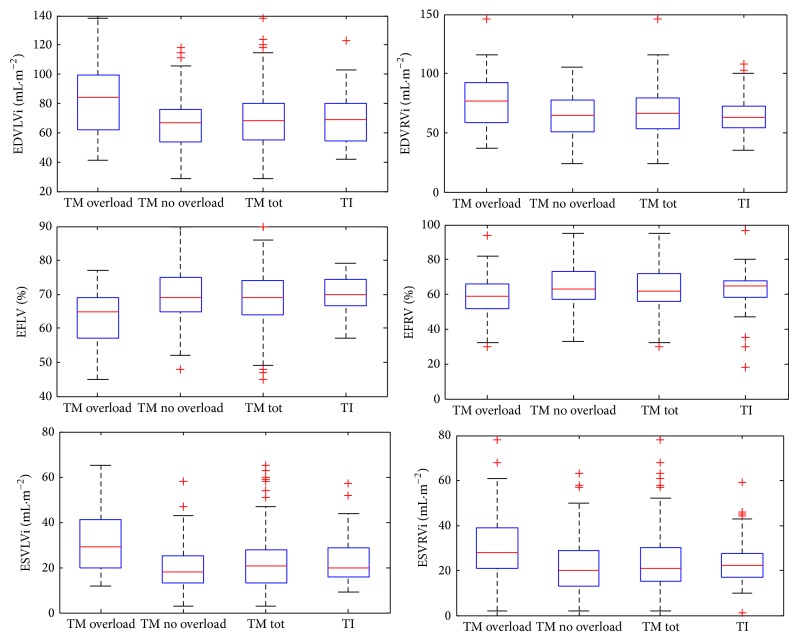
Boxplots of the EDVLVi, EDVRVi, EFLV, EFRV, SVLVi, and SVRVi of TM patients with iron overload, TM patients without iron overload, total of TM patients, and TI patients.

Significant differences were found between TM patients with iron overload and TM patients without iron overload: in TM patients with iron overload, decreased LV and RV ejection fraction and increased volumes (EDVLVi, ESVLVi, EDVRVi, and ESVRVi) were observed, compared to TM patients without iron overload. Comparing TM patients with iron overload to TI patients, we observed EFLV-EFRV values significantly lower in TM patients with respect to TI patients while, on the other hand, EDVLVi, ESVLVi, EDVRVi, and ESVRVi values were significantly higher in TM patients with respect to TI patients. Finally, no significant differences were found in the comparison between TM patients without iron overload and TI patients (Figure 3, Table 1).

## 4. Discussion

Cardiovascular involvement represents a well-known complication and the primary cause of mortality both in TM and in TI. Nowadays, iron overload related cardiomyopathy is the most frequent cause of heart failure in TM patients. Iron burden is related to a combination of repeated blood transfusion, with each unit of blood containing 200–250 mg of elemental iron, and excessive gastrointestinal adsorption. Transfusion iron is deposited in the reticuloendothelial system (RES); when the stores of the RES are saturated, iron deposition increases in parenchymal tissues: endocrine glands, hepatocytes, and myocardium are the most involved sites [20, 21]. Intracellular cardiac lysosome system stores the relatively nontoxic iron forms, hemosiderin and ferritin; however, once the storage capacity is exhausted, nontransferrin-bound iron, which is highly toxic, can be released with a consequent formation of hydroxyl radicals. In the heart, it determines an impaired function of respiratory chain, ventricular dysfunction, and the potential progression to heart failure [22, 23]. Left ventricular dysfunction and symptomatic heart failure usually develop at an advanced stage of cardiac siderosis. Cardiovascular involvement in TI is quite different: patients live longer and are usually transfusion-independent, at least for the first decades of their life. Haemoglobin levels are therefore lower and, compared to TM, a lower iron load is also maintained. Several factors have been reported to interfere in the pathophysiology of cardiovascular abnormalities in TI. These affect left and right heart, therefore leading to ventricular remodelling and heart failure [7, 11]. In this study, TI and TM patients without clinical signs or symptoms of cardiac failure or pulmonary hypertension were studied using CMR. 41 patients with a diagnosis of TM presented myocardial iron overload (*T*2^∗^ < 20 ms), although they were in chelation therapy. TI patients presented no myocardial overload (*T*2^∗^ > 20 ms). We evaluated the relationship between myocardial iron loading and LV-RV function, using myocardial *T*2^∗^ and functional CMR imaging (considered the gold standard for assessment of cardiac iron loading and cardiac function) [14]. The normal reference ranges of the LV-RV volumetric and functional parameters in thalassemia patients without cardiac iron load differ significantly from those of healthy nonanaemic controls. Thalassemia patients have greater LVEF and RVEF than healthy subjects [23–25], so we can say that thalassemia patients have left and right ventricular dysfunction at higher values of LVEF-RVEF than nonanaemic control [26, 27]. This is an important factor in the interpretation of the impaired ejection fraction. Our data showed a mirror effect on both RV and LV volumes and functions in TM patients: we found progressive enlargement and dysfunction with increasing myocardial siderosis. The RVEF and LVEF presented significant correlation. Apart from myocardial iron loading, we did not observe any clinical factors that could justify the observed effect on LV-RV function of TM patients. Anderson et al. described the progressive impairment in LVEF when *T*2^∗^ fell below 20 ms [13]. These findings have been confirmed by Tanner et al. [25] and by our data. We observed similar results in RV parameters: increasing myocardial siderosis can be associated with RV dysfunction and can be considered a significant contributor to heart failure in TM patients. Our data confirmed findings by Alpendurada et al. [27]. Comparing TM patients with iron overload and TI patients, we found significant differences between the two groups in terms of volumetric and functional parameters, while no significant differences were found between TM without cardiac siderosis and TI patients. According to our hypothesis, we demonstrated that TM patients can be differentiated from the TI group in relation to *T*2^∗^ values: this data is related to the different blood transfusion rate between the two populations and allows hypothesizing that in TM and TI patients the pathophysiologic mechanism of LV-RV dysfunction is different. In fact, although in TM patients the iron plays a crucial role in the development of heart failure, in TI the high output cardiac state seems more important. The high output state represents one of the basic pathophysiological mechanisms of cardiovascular involvement in these patients. Chronic anaemia, however, is not always severe in TI patients: in fact, haemoglobin levels usually range between 7 and 11 g/dL and are apparently not the only cause of a high output state in these patients. In normal subjects, resting cardiac output is kept within normal limits when haemoglobin levels range between 8 and 10 g/dL [28]. Besides the overall haemoglobin level, the proportions of the different haemoglobin subtypes, especially the high percentage of fetal haemoglobin (HbF), are also important. In particular, HbF reduces tissue oxygen delivery due to its increased oxygen affinity [29]. Thus, although the patient's haemoglobin concentration is close to normal values (11 g/dL), the most part of it could be HbF. Both chronic anemia and increased HbF percentage result in prolonged tissue hypoxia. This leads to a compensatory bone marrow expansion with extramedullary haemopoiesis, splenomegaly, and hepatomegaly, all of which also contribute to the high output state through peripheral vasodilatation and shunt development. Vessels in TI are also more susceptible to pulse pressure-driven dilatation due to a coexistent elastic tissue injury. The contribution of peripheral vasodilatation and intramedullary shunting could play an important role in the development of a high output state [7].

## 5. Conclusions

Our findings may have important implications in terms of patients' management. Identifying TM patients at risk of iron-overloaded cardiomyopathy is mandatory: this study demonstrated that myocardial iron load assessed by CMR is associated with deterioration of left and right ventricular function in TM patients. Until now there has been no agreement about the follow-up strategies and the therapeutic approach of TI patients. The absence of iron overload as the main cause of heart failure may offer a new concept in TI treatment. However, further studies are needed to identify the clinical presentation and the CMR findings according to genetic pattern of each TI subgroup.

## Figures and Tables

**Table 1:** Comparison between TM without myocardial iron overload (*T*2^∗^ > 20 ms) and TM with myocardial iron overload (*T*2^∗^ < 20 ms); TM with myocardial iron overload (*T*2^∗^ < 20 ms) and TI; TM without myocardial iron overload (*T*2^∗^ > 20 ms) and TI.

Parameter	Patients' group		*P* value	Patients' group		*P* value	Patients' group		*P* value
	*T*2^∗^ > 20 ms Mean (SD)	*T*2^∗^ < 20 ms Mean (SD)		TI Mean (SD)	TM (<20 ms) Mean (SD)		TI Mean (SD)	*T*2^∗^ > 20 ms Mean (SD)	
*n*	145	41	—	56	41	—	56	145	—
*T*2^∗^ ms	32.1 (5.7)	11 (8.1)	—	32.6 (4.7)	11 (8.1)	—	32.6 (4.7)	32.1 (5.7)	>0.05
EFLV %	70.1 (6.9)	62.8 (8.5)	<0.05	69.8 (5.7)	62.8 (8.5)	<0.05	69.8 (5.7)	70.1 (6.9)	>0.05
EFRV %	66.9 (8.9)	54.0 (8.6)	<0.05	67.2 (5.2)	54.0 (8.6)	<0.05	67.2 (5.2)	66.9 (8.9)	>0.05
EDVLVi mL/m^2^	66.6 (18)	82.0 (24)	<0.05	70.1 (17)	82.0 (24)	<0.05	70.1 (17)	66.6 (18)	>0.05
ESVLVi mL/m^2^	19.9 (9.6)	31.6 (15)	<0.05	22.3 (10)	31.6 (15)	<0.05	22.3 (10)	19.9 (9.6)	>0.05
SVLVi mL/m^2^	46.4 (11)	50.1 (12)	>0.05	48.1 (9.7)	50.1 (12)	>0.05	48.1 (9.7)	46.4 (11)	>0.05
massLVi g/m^2^	95.3 (27)	97.7 (25)	>0.05	99.8 (27)	97.7 (25)	>0.05	99.8 (27)	95.3 (27)	>0.05
EDVRVi mL/m^2^	64.1 (17)	77.4 (22)	<0.05	65.7 (18)	77.4 (22)	<0.05	65.7 (18)	64.1 (17)	>0.05
ESVRVi mL/m^2^	21.8 (12)	31.8 (16)	<0.05	23.8 (11)	31.8 (16)	<0.05	23.8 (11)	21.8 (12)	>0.05
SVRVi mL/m^2^	42.2 (10)	42.4 (13)	>0.05	41.4 (11)	42.4 (13)	>0.05	41.4 (11)	40.2 (10)	>0.05
massRVi g/m^2^	29.3 (18)	28.7 (19)	>0.05	30.1 (16)	28.7 (19)	>0.05	30.1 (16)	29.3 (18)	>0.05
